# Assessment of Estrogenic Endocrine-Disrupting Chemical Actions in the Brain Using *in Vivo* Somatic Gene Transfer

**DOI:** 10.1289/ehp.7418

**Published:** 2004-12-02

**Authors:** Vance L. Trudeau, Nathalie Turque, Sébastien Le Mével, Caroline Alliot, Natacha Gallant, Laurent Coen, Farzad Pakdel, Barbara Demeneix

**Affiliations:** ^1^Centre for Advanced Research in Environmental Genomics, Department of Biology, University of Ottawa, Ottawa, Ontario, Canada; ^2^UMR-5166, Evolution des Régulations Endocriniennes, Museum National d’Histoire Naturelle, Centre National de la Recherche Scientifique, Paris, France; ^3^UMR-6026, Equipe d’Endocrinologie Moléculaire de la Reproduction, Centre National de la Recherche Scientifique, Université de Rennes, Rennes, France

**Keywords:** bisphenol A, brain, estrogen response element, ethinylestradiol, goldfish, somatic gene transfer, *Xenopus laevis*

## Abstract

Estrogenic endocrine-disrupting chemicals abnormally stimulate vitellogenin gene expression and production in the liver of many male aquatic vertebrates. However, very few studies demonstrate the effects of estrogenic pollutants on brain function. We have used polyethylenimine-mediated *in vivo* somatic gene transfer to introduce an estrogen response element–thymidine kinase–luciferase (ERE-TK-LUC) construct into the brain. To determine if waterborne estrogenic chemicals modulate gene transcription in the brain, we injected the estrogen-sensitive construct into the brains of Nieuwkoop-Faber stage 54 *Xenopus laevis* tadpoles. Both ethinylestradiol (EE2; *p* < 0.002) and bisphenol A (BPA; *p* < 0.03) increased luciferase activity by 1.9- and 1.5-fold, respectively. In contrast, low physiologic levels of 17β-estradiol had no effect (*p* > 0.05). The mixed antagonist/agonist tamoxifen was estrogenic *in vivo* and increased (*p* < 0.003) luciferase activity in the tadpole brain by 2.3-fold. There have been no previous reports of somatic gene transfer to the fish brain; therefore, it was necessary to optimize injection and transfection conditions for the adult goldfish (*Carassius auratus*). Following third brain ventricle injection of cytomegalovirus (CMV)-green fluorescent protein or CMV-LUC gene constructs, we established that cells in the telencephalon and optic tectum are transfected. Optimal transfections were achieved with 1 μg DNA complexed with 18 nmol 22 kDa polyethylenimine 4 days after brain injections. Exposure to EE2 increased brain luciferase activity by 2-fold in males (*p* < 0.05) but not in females. Activation of an ERE-dependent luciferase reporter gene in both tadpole and fish indicates that waterborne estrogens can directly modulate transcription of estrogen-responsive genes in the brain. We provide a method adaptable to aquatic organisms to study the direct regulation of estrogen-responsive genes *in vivo*.

In both female and male vertebrates, estrogens affect many aspects of development, growth, sexual differentiation, and reproductive behavior. Estrogens also exert positive and negative feedback effects on the hypothalamopituitary axis to regulate the secretion of gonadotropic and other pituitary hormones (Hess 2003; Korach et al. 2003; McLachlan 2001; Trudeau 1997). Estrogens, notably 17β-estradiol (E_2_), are also involved in reproductive disorders such as breast and endometrial cancers (Feigelson and Henderson 1996; Graham et al. 2000). It is now recognized that there is worldwide contamination of water systems with chemicals and pharmaceuticals that mimic or inhibit estrogen action (Kolpin et al. 2002; Metcalfe et al. 2003; Ternes et al. 1999). The contraceptive steroid ethinylestradiol (EE2) and the natural hormone E_2_ are among the most commonly detected hormones in surface waters and effluents from sewage treatment plants (Ternes et al. 1999). E_2_ and EE2 were detected in effluents of sewage treatment plants in different countries at concentrations ranging up to 64 ng/L and 42 ng/L, respectively (Yin et al. 2002). The presence of these estrogens in Canadian sewage treatment plants has been documented with median concentrations of 9 ng/L for EE2 and 6 ng/L for E_2_ (Ternes et al. 1999). A recent study of 139 U.S. rivers reported maximum concentrations of 830 ng/L (~ 2.8 nM) for EE2 and 200 ng/L (~ 0.7 nM) for E_2_ (Kolpin et al. 2002).

The xenoestrogen bisphenol A (BPA) is primarily used in the production of poly-carbonate and epoxy resins and is found in many plastic products, including food can linings and dental sealants. The widespread industrial and household use, economic importance, and near ubiquitous presence of BPA in the environment (Lee and Peart 2000; Staples et al. 1998) emphasize its risk as an endocrine disruptor. Concentrations of BPA in surface waters have been reported to be, in the most severe cases, as high as 17,200 μg/L (~ 47 μM) in leachates from hazardous waste landfill sites (Yamamoto et al. 2001), but usually concentrations have been around or below 1 μg/L (~ 2.7 nM) (Belfroid et al. 2002). However, the concentration of BPA in many polluted lakes and rivers is not known.

A host of developmental and reproductive abnormalities in many species, including humans (Guillette et al. 1995; McLachlan 2001; Tyler et al. 1998), result from exposure to estrogenic endocrine-disrupting chemicals (EDCs). For example, octylphenol, BPA, and EE2 all stimulate abnormal production of the egg yolk protein vitellogenin in male fish (Arukwe 2001; Sumpter and Jobling 1995). Moreover, BPA induced testisova in medaka exposed to a concentration of 10 μg/L (~ 27 nM) (Metcalfe et al. 2001). Other studies showed that estrogenic EDCs cause sex reversal in frogs and feminization of secondary sex characteristics in fish (Arcand-Hoy and Benson 1998; Bogi et al. 2002; Mackenzie et al. 2003).

The diversity of structure and origin of the multitude of compounds currently known to bind to estrogen receptors (ER)- αand ER-β make it difficult to predict activities *in vivo* in vertebrate animals (Sanchez et al. 2002; Segner et al. 2003; Yoon et al. 2001). Large-scale screening for estrogenic activities by traditional physiologic and toxicologic methods is time-consuming and costly. A variety of effective *in vitro* ER binding assays and estrogen-responsive reporter systems in bacterial, yeast, and vertebrate cell systems have defined much of our understanding of estrogen and EDC actions (Ackermann et al. 2002; Matthews et al. 2002; Metivier et al. 2001, 2003; Petit et al. 1997; Zacharewski 1997). However, results derived *in vitro* for ER binding, hepatocyte vitellogenin induction, or ER reporter gene assays often do not always accurately reflect results obtained *in vivo* (Andersen et al. 1999; Segner et al. 2003). When E_2_ or estrogenic mimics bind to ERs, receptor dimerization and recruitment of transcriptional comodulators are initiated, and the hormone–receptor complex binds to the estrogen response element (ERE) and subsequently regulates transcription in an ordered and cyclic manner (Metivier et al. 2003; Robinson-Rechavi et al. 2003). Some of the discrepancies between *in vitro* assays and *in vivo* physiologic experiments may reflect the observations that ERα and ERβ differ dramatically in tissue and cellular distributions, biologic function (Abraham et al. 2004; Hess 2003; Korach et al. 2003), and their affinities for estrogenic chemicals (Le Guevel and Pakdel 2001; Yoon et al. 2001). Moreover, the likelihood that the availability of transcriptional comodulators of the ERs *in vitro* and *in vivo* is similar is highly unlikely (Graham et al. 2000), and thus, *in vitro* models cannot mimic the complexities of whole animal systems with respect to estrogen-dependent processes and responses to EDCs.

To begin to overcome some of the challenges of *in vivo* assessment of EDC modulation of gene transcription, we have validated polyethylenimine (PEI)-mediated somatic gene transfer (Lemkine and Demeneix 2001; Ouatas et al. 1998) to introduce an estrogen response element–thymidine kinase–luciferase (ERE-TK-LUC) construct into the intact brain. The effects of environmentally relevant concentrations of estrogenic pollutants on the expression of an established ERE reporter system characterized *in vitro* have been studied in several cell lines (Ackermann et al. 2002; Metivier et al. 2001). We have adapted somatic gene transfer procedures previously used for the *Xenopus laevis* tadpole (Ouatas et al. 1998) to demonstrate that waterborne estrogenic pollutants regulate transcription *in vivo*, both in *X. laevis* tadpoles and in the adult goldfish, *Carassius auratus*.

## Materials and Methods

### Plasmid constructs.

We used a consensus ERE with a minimal thymidine kinase promoter driving firefly luciferase activity (ERE-TK-LUC) as described previously (Metivier et al. 2001). This ERE reporter system is well characterized *in vitro* in several cell lines (Ackermann et al. 2002; Metivier et al. 2001) and responds to both zebrafish (Menuet et al. 2002) and goldfish ER-α and ER-β subtypes (Marlatt V, Trudeau VL, Moon TW, unpublished data). cytomegalovirus (CMV)-luciferase (CMV-LUC) and CMV-green fluorescent protein (CMV-GFP) were from Vical Inc. (San Diego, CA, USA) and Invitrogen (Carlsbad, CA, USA), respectively.

### Luciferase activity.

Brains from luciferase-transfected *X. laevis* tadpoles or goldfish were dissected and frozen in liquid nitrogen and stored at –80°C until assayed for luciferase activity [relative light units (RLUs)] according to the manufacturer’s instructions (Promega, Charbonnieres, France). Frozen brains were sonicated in ice-cold luciferase lysis buffer (200 μL for tadpoles, 500 μL for goldfish) and then centrifuged 10 min at 12,000*g* (4°C) to precipitate nonsoluble particles and proteins. Twenty microliters of the supernatant was mixed by vortexing with 100 μL luciferase substrate and counted immediately (10 sec) using a single-well luminometer as previously reported (Ouatas et al. 1998).

### Assessment of ERE-TK-LUC activity in the brains of X. laevis tadpoles.

Previous data have demonstrated that somatic gene transfer is an effective method to study thyroid hormone (TH) responses in the *X. laevis* tadpole (Ouatas et al. 1998). To avoid possible TH–E_2_ interactions in the brain (Dellovade et al. 1999), we used Nieuwkoop-Faber (NF) stage 54 *X. laevis* tadpoles (Nieuwkoop and Faber 1967) in which TH synthesis was inhibited by 1 g/L sodium perchlorate to determine whether waterborne estrogenic chemicals activate ERE-TK-LUC injected into the larval brain. In all cases, we report nominal water concentrations of estrogenic chemicals. In experiment 1, tadpoles were preexposed for 48 hr to 0.5 nM EE2, 5 nM E_2_, 50 nM BPA (bisphenol A methylacrylate; Sigma, St. Louis, MO, USA), or ethanol vehicle (0.4 mL in 4 L water in 10-L glass tanks; 20–22°C). In experiment 2, tadpoles were similarly preexposed to 200 nM tamoxifen (TAM; Sigma), a mixed ER antagonist/agonist. After the preexposure period, tadpoles were injected with ERE-TK-LUC (200 ng in 1 μL) complexed with 6 equivalents (eq) of 22 kDa polyethylenimine (PEI; Euromedex, Souffelweyersheim, France) in a 5% glucose solution into the brain as previously described (Ouatas et al. 1998) and returned to clean water freshly treated with estrogenic chemicals and exposed a further 48 hr. Animals were then sacrificed and whole brains dissected for determination of total luciferase activities.

### Development of a somatic gene transfer method for the goldfish brain.

All fish were purchased from a local supplier (Paris, France) and maintained at 20–22°C. Adult male and female goldfish were used to optimize *in vivo* transfer methods and to determine if water-borne estrogenic chemicals activate ERE-TK-LUC injected into the adult brain. First, we established the least intrusive method for injection into the forebrain region. Stereotaxic methods have been established for brain third ventricular injections of medium- to large-sized goldfish (25–35 g), which involved surgical opening of the cranium (Peter and Gill 1975). A modification of this method was used to inject CMV-GFP (800 ng in 2 μL; 6 eq of PEI) to determine the regions transfected by ventricular injections in adults. The skull was opened with fine scissors, and, rather than using a Hamilton syringe as originally reported (Peter and Gill 1975), we used a fine glass capillary held in a micromanipulator as reported for tadpoles (Ouatas et al. 1998). Animals were sacrificed 6 days after brain injections. Whole brain was dissected and first examined directly without fixation using epifluorescence microscopy (Olympus, Hamburg, Germany) to determine if GFP was being expressed. Some brains were fixed in 2% paraformalde-hyde in phosphate buffer and processed for standard cryostat sectioning as reported previously (Coen et al. 1999).

This surgical approach permits precise injection into the regions of interest but is slow, highly invasive, and not amenable to the treatment of large numbers of animals. We developed an alternative approach that involves only minor surgery and is more rapidly completed. Animals were anesthetized in 0.05% MS-222 and placed in a sponge holder. Under a dissection microscope and using a modeler’s drill apparatus (model 28-515; Proxxon, Niersbach, Germany) with a 0.5-mm bit attached, a small hole was made in the cranium at the midline 1–2 mm posterior to the posterior margins of the eye. In small goldfish (3–10 g), preliminary trials using 0.1% fast green dye (Sigma) established that an injection of 4 μL at an angle of approximately 45–50° relative to the top of the head and at a depth of 3–4 mm would partially fill the brain ventricle and expose cells in the forebrain and optic tectum to the injected solution. In a trial using CMV-LUC (4 μL of 500 ng DNA/μL; *n* = 5), approximately 80–90% of the total brain luciferase activity was found in the telencephalon and optic tectum, whereas the hypothalamus and cerebellum plus hindbrain had very low levels of transfection (data not shown).

To establish the concentration of PEI necessary for optimal transfection, small goldfish (3–10 g) were injected with 1 g CMV-LUC in 4 μL complexed with 0, 3, 6, and 9 eq of PEI in a 5% glucose solution. Briefly, as previously described for tadpoles (Ouatas et al. 1998), the required amount of PEI is calculated based on the fact that 1 μg DNA contains 3 nmol phosphate and that 1 μL 0.1 M PEI is equivalent to 100 nmol of amine nitrogen. Therefore, to condense 10 μg DNA with 6 eq of PEI, 180 nmol PEI (i.e., 1.8 μL of 0.1 M PEI) is required. We also performed a time-course study in which animals were injected with 1 μg CMV-LUC in 4 μL complexed with 6 eq of PEI, and whole brains dissected at 2, 12, 24, 48, and 96 hr. After dissection, whole brains were immediately frozen in liquid nitrogen and processed for luciferase activity as described above for tadpole brain.

### Effects of estrogenic chemicals on ERE-TK-LUC in goldfish brain.

For this experiment, we used small goldfish of both sexes (in 50–70 L glass tanks). Because these animals were in the early stages of seasonal gonadal redevelopment and could not be distinguished by external secondary sex characteristics, they were randomly assigned to each of the treatment groups. To determine whether waterborne estrogenic chemicals activate ERE-TK-LUC injected into the adult brain, groups of animals were preexposed for 48 hr to 10 nM E_2_, 10 nM EE2, or ethanol vehicle (0.1 mL/L water). After the preexpo-sure period, ERE-TK-LUC was injected as described above, and the fish were returned to water freshly treated with estrogenic chemicals and exposed a further 48 hr, at which time the water was changed again. The injected ERE-TK-LUC (1 μg DNA in 4 μL) was complexed with 6 eq of 22 kDa PEI in a 5% glucose solution. Whole brains were dissected at 96 hr after injection. Injections, exposures, and dissections were randomized over 3 days. At the time of dissection, body weights and sex of the animals were recorded.

### Statistical analysis.

The levels of luciferase activity (RLU) per whole *X. laevis* tadpole brain are expressed relative to mean expression levels per experiment (i.e., for the corrected RLU the mean equals 1). Goldfish injected with the ERE-TK-LUC construct varied in size (3–10 g), and therefore an additional correction was made based on milligrams of brain protein in the extracted luciferase fraction that was measured according to the manufacturer’s instructions (BioRad, Marnes-la-Coquette, France). Data were analyzed by one-way or two-way analysis of variance (ANOVA) or Student’s *t*-test as appropriate (SigmaStat, version 2.03; SPSS Inc., Chicago, IL, USA).

## Results

### Effects of estrogenic chemicals on ERE-TK-LUC activity in the brains of X. laevis tadpoles.

**Figure 1:**
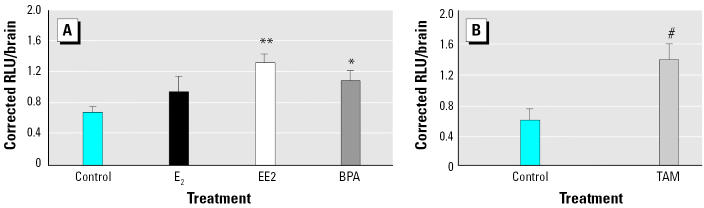
Effects of estrogenic chemicals on ERE-TK-LUC activity in the brains of perchlorate-treated NF stage 54 *X. laevis* tadpoles. (*A*) Effects of exposure to ethanol control (*n* = 18), E_2_ (5 nM; *n* = 14), EE2 (0.5 nM; *n* = 20), and BPA (50 nM; *n* = 19) on luciferase activity in the brains of tadpoles injected with ERE-TK-LUC (200 ng/μL; 6 eq of PEI); data are presented as mean ± SEM pooled from two separate exposures. (*B*) Effects of exposure to ethanol control (*n* = 11) and TAM (200 nM; *n* = 12) on luciferase activity in the brains of ERE-TK-LUC–injected tadpoles; data are presented as mean ± SEM.
*p < 0.03, **p < 0.002, and ^#^*p* < 0.003 compared with ethanol controls.

Figure 1A shows the effects of exposure to E_2_ (5 nM), EE2 (0.5 nM), and BPA (50 nM) on luciferase expression in the brains of ERE-TK-LUC–injected tadpoles. In this experiment the average activity (1 corrected RLU) represents approximately 73,000 RLU/brain. All data are expressed relative to this average. There was an effect of treatment (*p* < 0.004, one-way ANOVA) on luciferase activity. In the group treated with E_2_, mean levels were approximately 1.4-fold higher than in controls; however, this difference did not achieve statistical significance (*p* > 0.05). In contrast, EE2 induced a 1.9-fold increase (*p* < 0.002) in luciferase activity. Similarly, BPA also induced a 1.5-fold increase (*p* < 0.03) in luciferase activity measured in the whole brain. Figure 1B shows the effects of exposure to TAM (200 nM) on luciferase expression in the brains of ERE-TK-LUC–injected tadpoles. In this experiment the average activity (1 corrected RLU unit) represents approximately 38,000 RLU. All data are expressed relative to this average value. TAM induced a 2.3-fold increase (*p* < 0.003, *t*-test) in luciferase activity.

### Somatic gene transfer in the goldfish brain.

**Figure 2:**
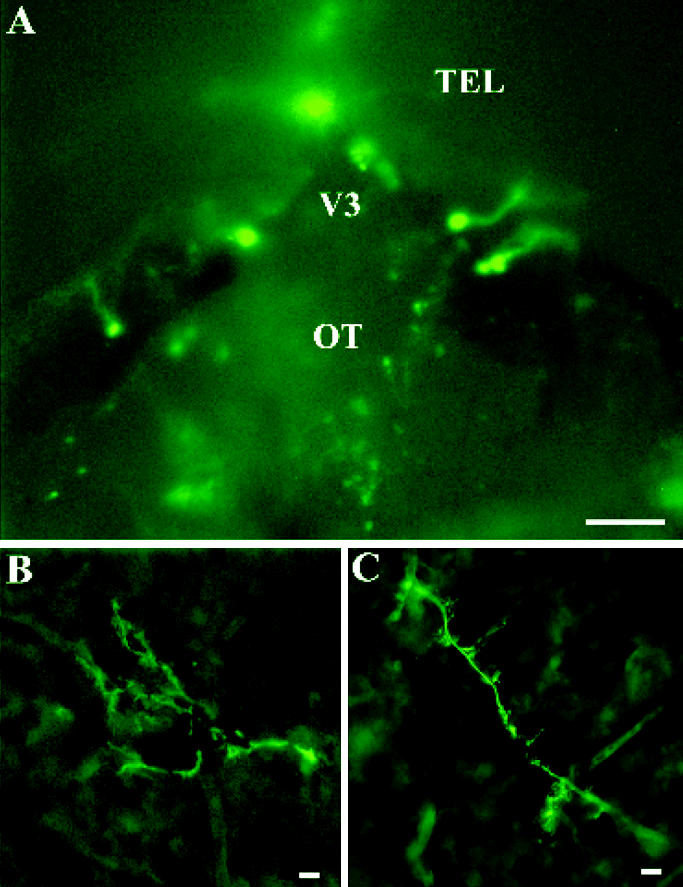
Expression of GFP in adult goldfish brain. (*A*) Expression of GFP in the telencephalon (TEL) and optic tectum (OT) of freshly dissected intact brain. Note the high expression around the brain third ventricle (V3); bar = 100 μm. (*B*) Sagittal section (25 μm) through the telencephalon of a goldfish showing a highly branching neuron expressing GFP throughout. The third ventricle is to the right; bar = 5 μm. (*C*) Sagittal section (25 μm) through the telencephalon of a goldfish showing a neuron extending dorsolaterally. The cell body (not easily visualized) is toward the top left corner; bar = 5 μm.

When we injected directly into the brain third ventricle of medium sized fish, cells in the telencephalon, optic tectum, and occasionally in the hypothalamus (not shown) were trans-fected with CMV-GFP (800 ng in 2 μL). Figure 2A shows the general distribution of GFP-expressing cells in a freshly dissected whole brain. Cells in the telencephalon close to the midline and brain third ventricle, as well as some cells in the optic tectum, were visualized with epifluorescence microscopy. Examples of two neurons expressing GFP are shown in Figure 2B and C. GFP was expressed in the cell body and also extensively in neuronal processes extending laterally away from the ventricular wall (represented by the border between Figure 2B,C). Note also that synaptic boutons and dendrites are also labeled with GFP. Cells in the nucleus preopticus peri-ventricularis and nucleus preopticus (Peter and Gill 1975) also expressed GFP (not shown).

**Figure 3:**
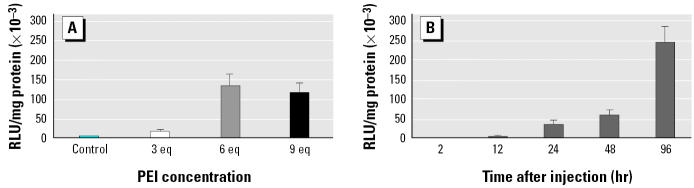
Optimization of PEI-based gene transfer in the goldfish brain. (*A*) Comparison of the efficiencies of 22 kDa linear PEI used at different ratios of PEI amines to DNA anions. Animals were injected with CMV-LUC DNA (1 μg in 4 μL) complexed with 0 (*n* = 9), 3 (*n* = 10), 6 (*n* = 10), and 9 (*n* = 10) eq of PEI; brains were dissected at 48 hr postinjection; and luciferase activity (RLU/mg protein × 10^–3^; mean ± SEM) was determined. (*B*) Time course of expression of CMV-LUC in the goldfish brain. Animals were injected with CMV-LUC DNA (1 μg in 4 μL) complexed with 6 eq of PEI; brains were dissected at 2 hr (*n* = 10), 12 hr (*n* = 10), 24 hr (*n* = 10), 48 hr (*n* = 7), and 96 hr (*n* = 5) postinjection, and luciferase activity (RLU/mg protein × 10^–3^; mean ± SEM) was determined.

Figure 3A illustrates the effect of PEI concentrations on transfection efficiency in the goldfish brain. Whereas 3 eq of PEI was only minimally effective, 6 eq of PEI produced maximal luciferase expression at 48 hr after brain injections. There was no further enhancement of transfection using 9 eq of PEI. Using 6 eq of PEI to complex CMV-LUC, a time-course analysis (Figure 3B) was performed. The highest luciferase expression was 96 hr after brain injection.

### Effects of estrogenic chemicals on ERE-TK-LUC in goldfish brain.

**Figure 4:**
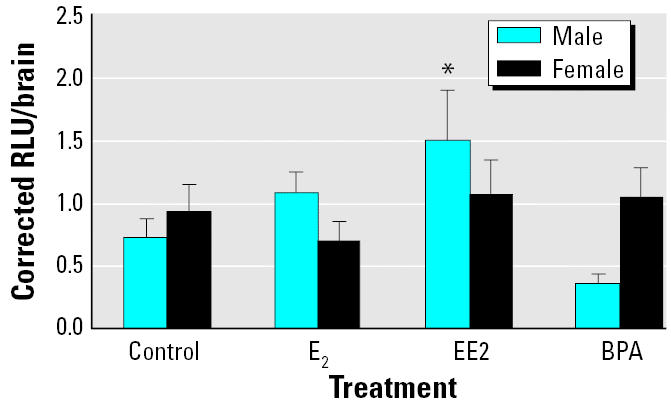
Effects of estrogenic chemicals on ERE-TK-LUC activity in the brains of male and female goldfish preexposed for 48 hr to E_2_ (10 nM; *n* = 14 males and 14 females), EE2 (10 nM; *n* = 8 males and 8 females), or ethanol vehicle (0.1 mL/L water; *n* = 8 males and 16 females). Data are presented as mean ± SEM.
*p < 0.05 compared with the male control values. levels

After having established a method for injection of DNA into the gold-fish brain (Figures 2 and 3), we examined the effects of E_2_, EE2, and BPA in small female and male goldfish. Figure 4 shows the effects of exposure to E_2_ (10 nM), EE2 (10 nM), and BPA (100 nM) on luciferase expression in the brains of ERE-TK-LUC–injected females and males. In this experiment the average activity (1 corrected unit) represents approximately 15,000 RLU/mg protein. All data are expressed relative to this average value. The effects of the various treatments on luciferase activity was dependent on the sex of the fish (two-way ANOVA: sex × treatment, *p* < 0.019). Basal luciferase activity was similar in control females and males (*p* > 0.05). In males treated with E_2_, mean levels were approximately 1.5-fold higher than in controls; however, this change was not statistically significant (*p* > 0.05). Additionally, E_2_ did not affect (*p* > 0.05) luciferase activity in females. In contrast, waterborne EE2 induced a 2-fold increase (*p* > 0.05) in luciferase activity in the male brain but had no effect in females (*p* > 0.05). Moreover, BPA did not affect (*p* > 0.05) luciferase activity in either sex.

## Discussion

Our results indicate that waterborne estrogenic chemicals can modulate brain activity in aquatic vertebrates. Using somatic gene transfer into the brains of tadpoles and adult fish, we showed that estrogenic chemicals activate expression of an introduced ERE-TK-LUC construct. This required adaptation of somatic gene transfer methods previously used in *X. laevis* (Ouatas et al. 1998) and mice (Guissouma et al. 1998) to study TH-driven gene expression and in *Xenopus tropicalis* (Rowe et al. 2002) to analyze apoptosis during metamorphosis. To our knowledge, PEI-mediated somatic gene transfer into the fish brain has not been previously reported. Optimal transfections were achieved with 1 μg DNA complexed with 18 nmol 22 kDa PEI 4 days after brain injections. However, longer time periods were not analyzed, and it is possible that expression in the adult goldfish brain would increase after 96 hr.

The potent estrogen from female contraceptives, EE2, and the natural estrogen E_2_ are found at picomolar to nanomolar concentrations in both European and North American sewage effluents and surface waters (Kolpin et al. 2002; Metcalfe et al. 2003; Ternes et al. 1999). We showed that short-term exposure to 0.5 nM EE2 in tadpoles and 10 nM EE2 in male goldfish increased the activity of a known estrogen-responsive reporter gene construct by approximately 2-fold. In contrast, female goldfish were not responsive to 10 nM water-borne EE2. The plasticizing agent BPA and the mixed ER antagonist/agonist TAM were both estrogenic in tadpole brain. In both male and female goldfish, high levels (100 nM) of BPA did not activate the estrogen-responsive reporter gene construct injected into the brain.

Although we did not directly compare transfection efficiencies in tadpoles and gold-fish, there appears to be an important difference. Based on the number of GFP-positive cells and the basal levels of luciferase expression, transfection appears less efficient in adult gold-fish compared with larval tadpole brain (Ouatas et al. 1998). The maximum activity of reporter gene luciferase from a whole brain per milligram of protein showed that transfection is ~ 30-fold more efficient in the tadpole compared with the adult goldfish. The reasons for this are unknown but likely relate to differences in injection methods, ratio of brain volume to injection volume, and/or cellular characteristics of larval versus adult brain. Results in goldfish are, however, similar to those obtained with PEI-mediated transfection of hypothalamic neurons of neonatal mice with the same CMV-LUC construct (Guissouma et al. 1998). Our results showed that cells in the adult goldfish forebrain and optic tectum are transfectable *in vivo*. Autoradiographic (Kim et al. 1978), immunocytochemical (Navas et al. 1995), and *in situ* hybridization (Menuet et al. 2002) studies showed that both ER-α and ER-β are expressed in the telencephalon and hypothalamus and especially in the preoptic area of fish. Using reverse-transcriptase polymerase chain reaction (RT-PCR), Choi and Habibi (2003) also detected both ERs in goldfish brain. Our results showed that *in vivo* transfection in the goldfish telencephalon can be used to study the regulation of ERE-driven expression by an estrogenic pollutant.

In both animal models, there was a relatively high basal luciferase activity in controls. This is likely due to two interacting factors: high *in vivo* activity of the minimal thymidine kinase promoter and effects of endogenous neuroestrogen on basal expression of the ERE-TK-LUC gene construct. In *X. laevis* tadpoles, estrogen production in the brain has not been studied, but at NF stage 54, whole-body E_2_ levels are easily detectable despite having declined relative to very high levels in early stages of development (Bogi et al. 2002). Male and female gonads are distinguishable by gross morphologic characteristics at NF stage 56 (Bogi et al. 2002). Therefore, it is likely that our tadpoles were producing endogenous estrogen. Relatively high basal ERE-TK-LUC activity at this stage of tadpole development suggests that ERs are active and/or that endogenous E_2_ is being produced and delivered to the transfected cells. The goldfish brain has a remarkable capacity to produce E_2_ from testosterone because of very high aromatase activity (Callard et al. 2001; Pasmanik and Callard 1988). The dose of E_2_ we used is within the physiologic range and thus would be unlikely to raise brain E_2_ above endogenous brain E_2_ concentrations, especially in females. It is known that EE2 is more potent that E_2_ in several assay systems using the same ERE-TK-LUC reporter gene (Ackermann et al. 2002; Le Guevel and Pakdel 2001). In female goldfish, 10 nM EE2 did not affect luciferase expression, similar to what was observed with E_2_. This is in contrast to males where EE2 induced a 2-fold increase in activity. We have previously observed marked sex differences in gold-fish neuroendocrine responses to sex steroids (Bosma et al. 2001). For example, whereas testosterone inhibited the expression of glutamic acid decarboylases (GAD65 and GAD67) in the telencephalon of sexually mature males, it was without effect in females (Lariviere K, Trudeau VL, unpublished data).

Our results indicate that short-term exposure to environmentally relevant water levels of BPA (50 nM, ~ 18 μg/L) can activate the ERE-TK-LUC construct in the tadpole brain. In contrast to effects in fish (Metcalfe et al. 2001; Staples et al. 1998), the effects of BPA in amphibians are not well studied. Kloas et al. (1999), using a static renewal exposure protocol, reported that BPA has estrogenic activity at 2.3 μg/L (~ 6.3 nM) and induces female-biased sex reversal in *X. laevis*. In a second study, the same researchers found that 100 nM BPA induced female-biased sex reversal in *X. laevis* (Levy et al. 2004). However, in a flow-through exposure system (Pickford et al. 2003), there were no observable effects of a range of BPA concentrations (0.83–497 μg/L; ~ 2.3 nM–1.4 μM) on larval growth, development, or sexual differentiation of *X. laevis* tadpoles. High, nonenvironmental of BPA (10–25 μM; 3,644–9,110 μg/L) have both teratogenic and antimetamorphic actions in *X. laevis* (Iwamuro et al. 2003), suggesting interference with the thyroid system. It is difficult at present to reconcile the different conclusions concerning the estrogenicity of BPA in frogs. However, given that BPA is continually being added to aquatic ecosystems through industrial and sewage effluent discharges and activates a known ERE–reporter gene construct in tadpole brain, it is a contaminant of environmental concern.

Activation of an ERE-dependent luciferase reporter gene in both tadpole and fish indicates that waterborne estrogens can directly modulate transcription of estrogen-responsive genes in the brain. Previous work from our laboratory demonstrated that environmentally relevant levels of the estrogenic pollutant octylphenol modulates the expression of multiple hypothalamic genes in leopard frog tadpoles (Crump et al. 2002) and in hatchling snapping turtles (Trudeau et al. 2002). In the latter study, differential display PCR was used, and it is not known if the affected transcripts were directly or indirectly regulated by 4-*t*-octylphenol or E_2_. As quantified in these latter studies using reverse Northern blotting, changes in several hypothalamic mRNAs induced by waterborne environmentally relevant levels of octylphenol in these studies were approximately 2-fold. This level of gene expression is similar to what we observed with ERE-dependent luciferase induction after EE2, BPA, and TAM exposures.

In this article we provide a method to study the direct regulation of estrogen-responsive genes *in vivo* in tadpoles and fish. The power of this approach is that it is possible to determine whether an estrogenic chemical is acting on a certain tissue. The ERE-dependent luciferase reporter gene is injected at a specific site and is responsive to the known nuclear ER subtypes (Menuet et al. 2002). Moreover, because the technique is based on the transcriptional mechanism of action of estrogen, a positive effect of a given chemical can be interpreted as activation of the ER. A somatic gene transfer technique may be generalized to other aquatic species, because it is easier and less time-consuming than identifying ER-regulated genes and their promoters in each species. The main limitation of the somatic gene transfer method described is that it is unlikely to detect indirect and nongenomic effects of estrogenic chemicals.
